# Supplementary material for: Impact of CO2 on Pyrolysis Products of Bituminous Coal and Platanus Sawdust
Source: Polymers (Basel). 2019 Aug 20;11(8):1370. doi: 10.3390/polym11081370 (PMC6722873; doi:10.3390/polym11081370)
Supplement: Supplementary file 1 [file polymers-11-01370-s001.pdf]

# Supplementary Materials

## Impact of $CO_2$ on bituminous coal and platanus sawdust pyrolysis

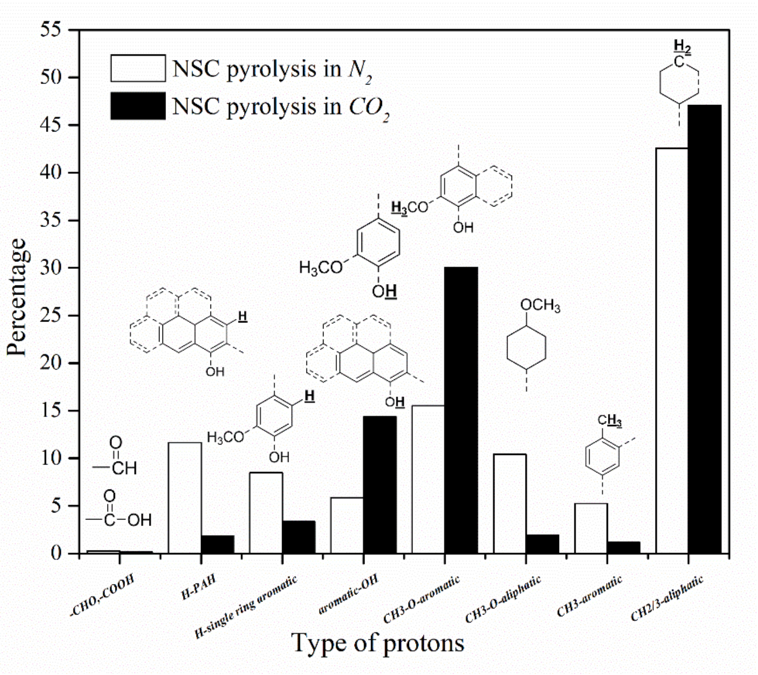


(**a**)

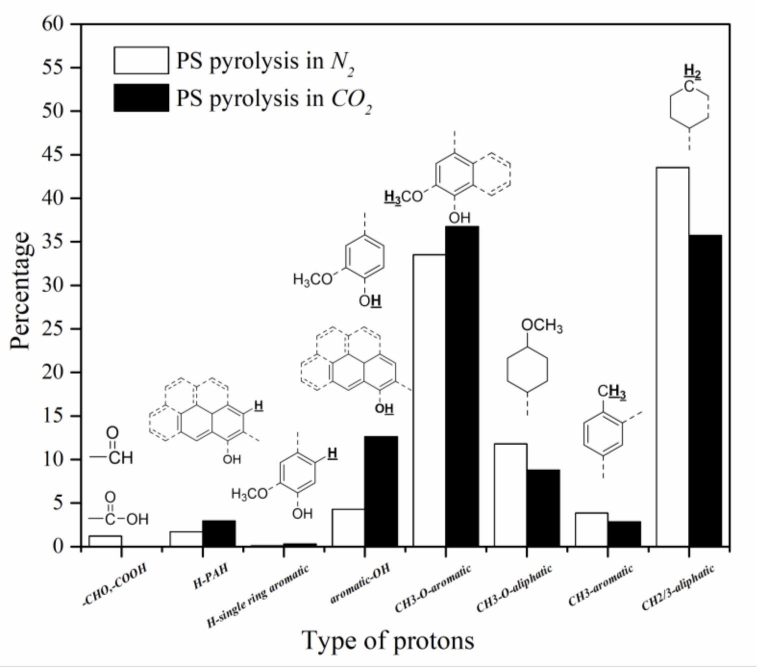


(**b**)

**Figure S1.** Comparison of the $^{1}$H NMR integration data of tar under $N_2$ atmosphere and $CO_2$ atmosphere: (**a**) From the pyrolysis of NSC; (**b**) From the pyrolysis of PS.

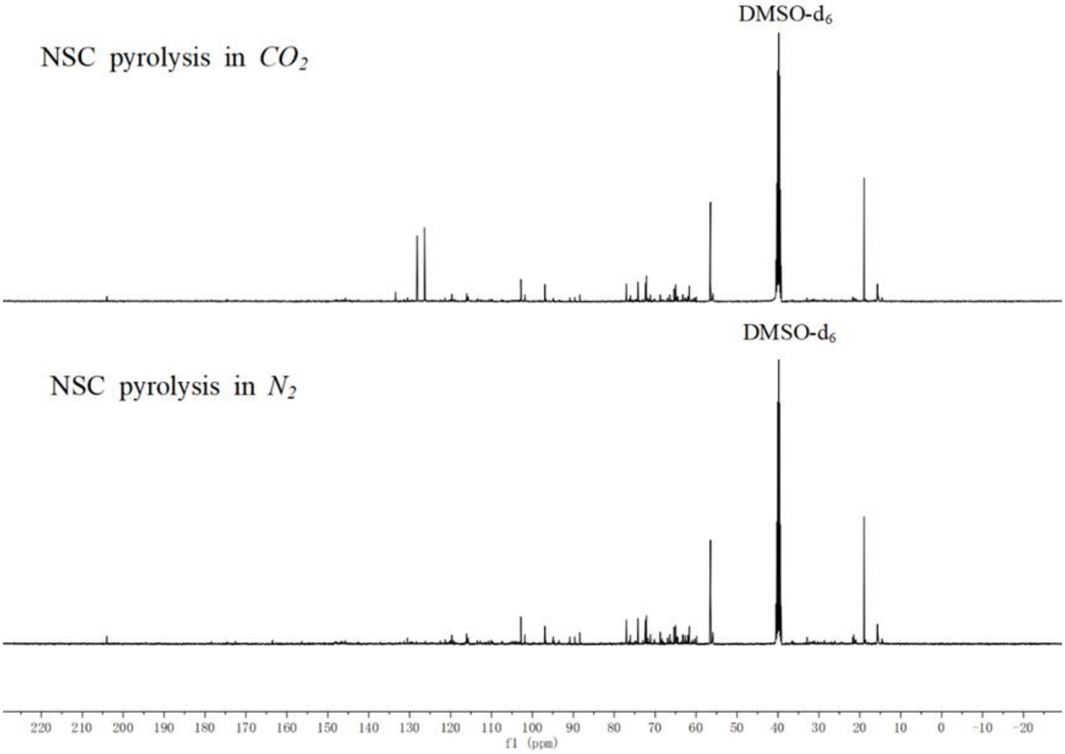


**Figure S2.** $^{13}$C NMR spectra of NSC tar under $N_2$ atmosphere and $CO_2$ atmosphere.

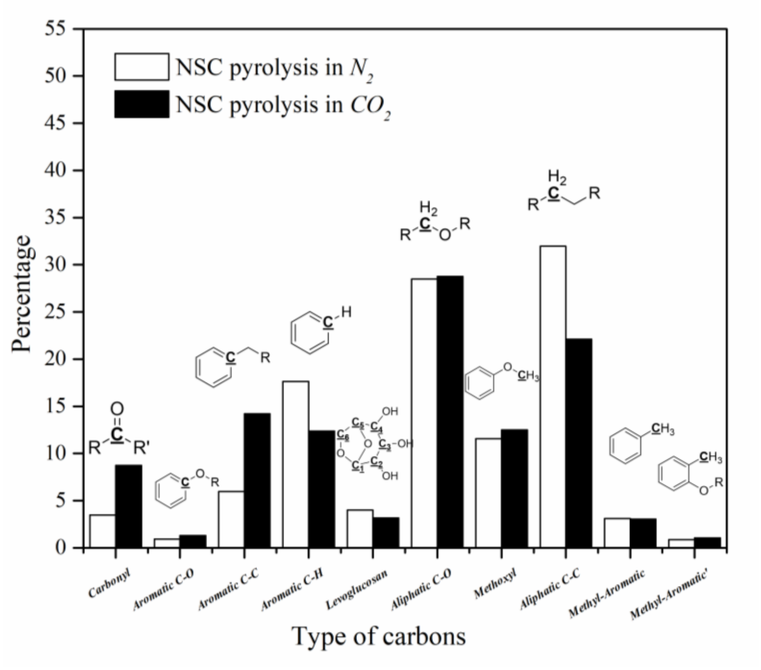


(**a**)

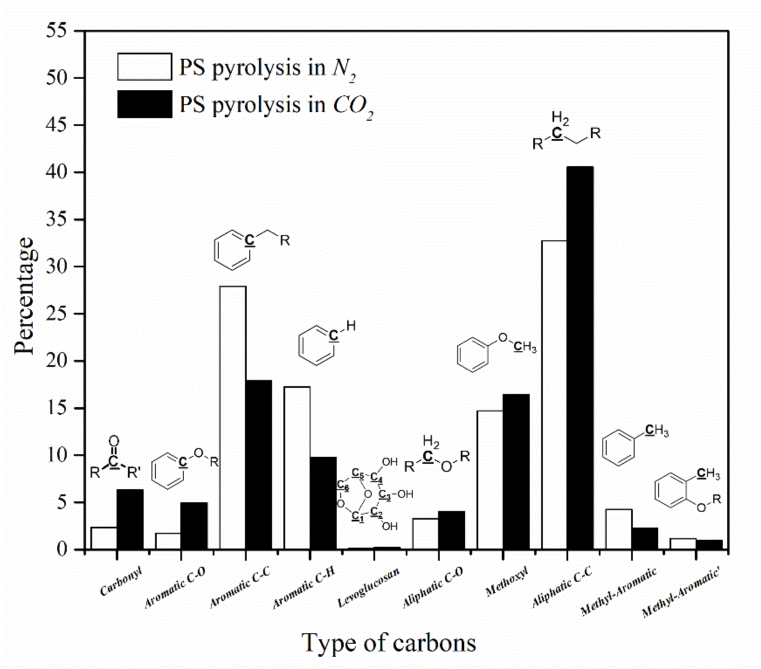


(**b**)

**Figure S3.** Comparison of the $^{13}C$ NMR integration data of tar under $N_2$ atmosphere and $CO_2$ atmosphere: (**a**) From the pyrolysis of NSC; (**b**) From the pyrolysis of PS.
